# Free breathing three-dimensional cardiac quantitative susceptibility mapping for differential cardiac chamber blood oxygenation – initial validation in patients with cardiovascular disease inclusive of direct comparison to invasive catheterization

**DOI:** 10.1186/s12968-019-0579-7

**Published:** 2019-11-18

**Authors:** Yan Wen, Jonathan W. Weinsaft, Thanh D. Nguyen, Zhe Liu, Evelyn M. Horn, Harsimran Singh, Jonathan Kochav, Sarah Eskreis-Winkler, Kofi Deh, Jiwon Kim, Martin R. Prince, Yi Wang, Pascal Spincemaille

**Affiliations:** 1 Meinig School of Biomedical Engineering, Cornell University, Ithaca, NY USA; 2 Department of Radiology, Weill Cornell Medicine, New York, NY USA; 3 Department of Medicine, Weill Cornell Medicine, New York, NY USA; 4 Weill Cornell Medical College, 515 East 71th Street, S101, New York, NY 10021 USA

**Keywords:** Quantitative susceptibility mapping, Oxygenation, Cardiac magnetic resonance

## Abstract

**Background:**

Differential blood oxygenation between left (LV) and right ventricles (RV; ΔSaO_2_) is a key index of cardiac performance; LV dysfunction yields increased RV blood pool deoxygenation. Deoxyhemoglobin increases blood magnetic susceptibility, which can be measured using an emerging cardiovascular magnetic resonance (CMR) technique, Quantitative Susceptibility Mapping (QSM) – a concept previously demonstrated in healthy subjects using a breath-hold 2D imaging approach (2D_BH_QSM). This study tested utility of a novel 3D free-breathing QSM approach (3D_NAV_QSM) in normative controls, and validated 3D_NAV_QSM for non-invasive ΔSaO_2_ quantification in patients undergoing invasive cardiac catheterization (cath).

**Methods:**

Initial control (*n* = 10) testing compared 2D_BH_QSM (ECG-triggered 2D gradient echo acquired at end-expiration) and 3D_NAV_QSM (ECG-triggered navigator gated gradient echo acquired in free breathing using a phase-ordered automatic window selection algorithm to partition data based on diaphragm position). Clinical testing was subsequently performed in patients being considered for cath, including 3D_NAV_QSM comparison to cine-CMR quantified LV function (*n* = 39), and invasive-cath quantified ΔSaO_2_ (*n* = 15). QSM was acquired using 3 T scanners; analysis was blinded to comparator tests (cine-CMR, cath).

**Results:**

3D_NAV_QSM generated interpretable QSM in all controls; 2D_BH_QSM was successful in 6/10. Among controls in whom both pulse sequences were successful, RV/LV susceptibility difference (and ΔSaO_2_) were not significantly different between 3D_NAV_QSM and 2D_BH_QSM (252 ± 39 ppb [17.5 ± 3.1%] vs. 211 ± 29 ppb [14.7 ± 2.0%]; *p* = 0.39). Acquisition times were 30% lower with 3D_NAV_QSM (4.7 ± 0.9 vs. 6.7 ± 0.5 min, *p* = 0.002), paralleling a trend towards lower LV mis-registration on 3D_NAV_QSM (*p* = 0.14). Among cardiac patients (63 ± 10y, 56% CAD) 3D_NAV_QSM was successful in 87% (34/39) and yielded higher ΔSaO_2_ (24.9 ± 6.1%) than in controls (*p* < 0.001). QSM-calculated ΔSaO_2_ was higher among patients with LV dysfunction as measured on cine-CMR based on left ventricular ejection fraction (29.4 ± 5.9% vs. 20.9 ± 5.7%, p < 0.001) or stroke volume (27.9 ± 7.5% vs. 22.4 ± 5.5%, *p* = 0.013). Cath measurements (*n* = 15) obtained within a mean interval of 4 ± 3 days from CMR demonstrated 3D_NAV_QSM to yield high correlation (r = 0.87, p < 0.001), small bias (− 0.1%), and good limits of agreement (±8.6%) with invasively measured ΔSaO_2_.

**Conclusion:**

3D_NAV_QSM provides a novel means of assessing cardiac performance. Differential susceptibility between the LV and RV is increased in patients with cine-CMR evidence of LV systolic dysfunction; QSM-quantified ΔSaO_2_ yields high correlation and good agreement with the reference of invasively-quantified ΔSaO_2_.

## Introduction

Differential blood oxygenation between the left and right heart (ΔSaO_2_) is an established index of cardiac performance; left ventricular (LV) dysfunction results in stagnant blood flow – resulting in increased time for organ extraction of oxygen from blood and delivery of a greater fraction of deoxygenated blood to the right heart. Increased ΔSaO_2_ has been shown to predict adverse prognosis in patients with heart failure with and without pulmonary hypertension [1–3] for whom it is commonly used to guide management [4, 5]. However, in current clinical practice, oxygen saturation is measured by invasive catheterization (cath). Non-invasive imaging methods to measure oxygenation in the heart are limited, prohibiting non-invasive quantification of cardiac blood pool oxygenation as part of routine clinical evaluation [6–13]. Given the fact that invasive catheterization entails procedural risks and can be challenging in critically ill patients [14–16], a non-invasive imaging method to accurately measure cardiac oxygenation would be of substantial clinical utility.

Quantitative susceptibility mapping (QSM) is an emerging cardiovascular magnetic resonance (CMR) technique that enables quantification of diamagnetic and paramagnetic materials [17–23]. Iron is a magnetically active element contained in hemoglobin that is central to oxygen transport - it is weakly diamagnetic when bound to oxygen, and paramagnetic when deoxygenated [24]. This change in magnetic susceptibility by deoxyheme [25, 26], provides a metric by which QSM can measure blood oxygen saturation. Prior work has validated QSM tissue characterization, including liver and brain iron content [22, 27–29]. Regarding blood oxygenation, a pilot study by our group showed cardiac QSM to be feasible in healthy subjects [30]. However, a 2D acquisition strategy was employed, which is suboptimal for imaging patients in whom breath-holding is often compromised. To address this, a free-breathing 3D QSM approach was developed that uses diaphragmatic navigator gating to track respiratory position. This study compared 3D free-breathing QSM (3D_NAV_QSM) to 2D breath held QSM (2D_BH_QSM) in controls, as well as to the reference of ΔSaO_2_ measured in patients undergoing invasive cardiac catheterization.

## Methods

### Study population

3D_NAV_QSM was first compared to 2D_BH_QSM among healthy subjects without clinically reported cardiovascular conditions or associated risk factors to test the relative performance in a cohort able to undergo prolonged imaging inclusive of both pulse sequences. Next, a second group of healthy subjects were scanned and rescanned to test the reproducibility of 3D_NAV_QSM ΔSaO_2_ measurement. The second group of healthy subjects was asked to get off and then get back on the table between the two scans. All healthy subjects were without self-reported cardiovascular disease or atherosclerosis risk factors.

After initial testing, 3D_NAV_QSM was then performed among clinical patients who were being considered for or had undergone invasive catheterization to quantify blood oxygen saturation. Catheterization was performed by experienced physicians using standard techniques; intracardiac blood samples were obtained under baseline conditions (without supplemental O_2_) and used to calculate ΔSaO_2_ between the left and right heart. To test the effect of gadolinium in ΔSaO_2_ measurement, QSM were obtained in 3 healthy subjects and in 4 patients both pre-contrast and ~ 30 min post-contrast administration.

This study was performed at Weill Cornell Medicine (WCM; New York, New York, USA). All participants (controls and patients) provided written informed consent for research participation. This protocol was performed with the approval from the WCM Institutional Review Board.

### Data acquisition

CMR was performed using commercial 3 T scanners (750/SIGNA, General Electric Healthcare, Waukesha Wisconsin, USA). 3D_NAV_QSM and 2D_BH_QSM imaging parameters were identical between healthy subjects and patients: 1^st^TE/ΔTE/#TE/TR/BW = 2.3 ms/3.6 ms/5/20 ms/111.1 kHz, acquisition matrix = 192 × 144, slice thickness = 5 mm, views per heartbeat = 10, parallel imaging factor = 2. Full 3D flow compensation was implemented for both 2D_BH_QSM and 3D_NAV_QSM to minimize the phase generated by intra-chamber blood flow [26]. To shorten 3D_NAV_QSM scan time, 75% partial Fourier acquisition was applied in the phase and slice encoding direction. Typical resolution is 1.5 × 1.5x5mm^3^, 40cm^2^ FOV, and 20 slices per scan. Furthermore, 2D_BH_QSM used electrocardiographic (ECG) gating and breath-holding, and 3D_NAV_QSM used ECG and respiratory gating to ensure the acquisition of data at a consistent cardiac and respiratory phase. QSM was performed using non-contrast CMR in healthy subjects, and at the end of clinical exams in patients, which was approximately ~ 30 min post-gadolinium (Dotarem [gadoterate meglumine]; 0.2 mmol/kg) infusion. As stated above, in 3 healthy subjects and 4 patients, QSM was acquired both pre- and post-contrast.

2D_BH_QSM employed a conventional ECG-triggered multi-echo gradient echo sequence, for which data was acquired during end-expiration (~ 12 s per breath-hold). 3D_NAV_QSM employed a tailored ECG-triggered navigator gated multi-echo gradient echo sequence, for which data was acquired during free breathing: a cross pair diaphragmatic navigator was used to track respiratory motion [31, 32]. A 2-bin phase-ordered automatic window selection (PAWS) gating algorithm (4 mm effective gating window) was used to tailor data acquisition according to diaphragm position in real-time [22]. In PAWS, each diaphragm position falls within a 2 mm bin for which k-space is acquired from alternating directions from bin to bin. The scan is complete when two adjacent bins have acquired all necessary k-space lines. Two navigator echoes were used in each heartbeat. The first navigator was acquired immediately before acquisition and used for PAWS gating. The second navigator was acquired immediately after data acquisition and used to provide additional motion suppression: if the difference between diaphragm positions detected by the two navigator echoes was > 4 mm for a given heartbeat, then the data acquired in that heartbeat were discarded and scheduled to be reacquired in a later heartbeat.

Ancillary imaging was performed to test QSM in relation to conventional cardiac functional/ remodeling indices. Cine-CMR was performed using a conventional balanced steady state free precession (bSSFP) pulse sequence with typical parameters: TE = 1.4 ms, TR = 3.8 ms, FA = 60^o^, bandwidth = 781 Hz/Px, resolution: 1.5 × 1.5x6mm^3^, and SENSE acceleration R = 2, which was acquired in contiguous long and short axis images, the latter of which were segmented to quantify LV end-diastolic and end-systolic chamber volumes for calculation of LV and right ventricular (RV) ejection fraction (EF) as well as stroke volume.

### QSM post processing

QSM maps were reconstructed by first obtaining a total field map (containing both the local and background field) with the contributions of fat chemical shift removed. This was done using both graph-cut based phase unwrapping [33] and IDEAL water/fat separation [34] with iterative chemical shift update [35]. Next, a susceptibility map was obtained using the preconditioned total field inversion method [36]. In this work, two regularization terms, similar to the regularization terms described in MEDI+ 0 [37], were added to the inversion to restrict the susceptibility variations within the RV and LV as follows:

1 $$ {y}^{\ast }=\underset{y}{\mathrm{argmin}}{\displaystyle \begin{array}{c}\frac{1}{2}{\left\Vert w\left(f-d\otimes Py\right)\right\Vert}_2^2+\lambda {\left\Vert {M}_G\nabla Py\right\Vert}_1\\ {}+{\lambda}_{RV}{\left\Vert {M}_{RV}P\left(y-{\overline{y}}^{RV}\right)\right\Vert}_2^2+{\lambda}_{LV}{\left\Vert {M}_{LV}P\left(y-{\overline{y}}^{LV}\right)\right\Vert}_2^2\end{array}} $$

The first two terms are the data fidelity term and structure consistency regularization term, respectively, where *w* is the signal to noise (SNR) weighting, *f* is the total field, *d* is the dipole kernel, * is the convolution operator, *P* is the preconditioner, *λ* is the regularization parameter, *M*_*G*_ is a binary edge mask constructed by retaining the highest 70% of gradients of the T2*w image obtained by taking the square root of the sum of squares gradient recalled echo (GRE) images across echoes), and ∇ is the gradient operator [20], *P* is a binary mask that is 1 inside the region of interest (ROI) and a larger value, *P*_*outside*_, outside of the ROI (see below). The final QSM map, *χ*, is then *χ* = *Py*^∗^. The last two terms constrain the susceptibility variation within the RV and the LV blood pools, where *λ*_*RV*_ and *λ*_*LV*_ are the regularization parameters, *M*_*RV*_ and *M*_*LV*_ are the mask for RV and LV obtained through manual segmentation on the GRE images. ${\overline{\ y}}^{RV}$ and ${\overline{\ y}}^{LV}$ are the average susceptibility over the RV and LV blood pools, respectively. In this study, the values of *P*_*outside*_ = 20, *λ* = 1/1000, and *λ*_*RV*_ = *λ*_*LV*_ = 1/20 were empirically determined in an initial study in healthy subjects via visual inspection of the corresponding QSM, and then fixed for subsequent subjects.

To account for potential field errors from water/fat separation, an iterative reweighted least squares fitting method, MERIT [38], was implemented to modify noise weighting of fat voxels (fat fraction> 30%) in each Gauss-Newton iteration to account for residual fat chemical shift not removed from the total field in the water/fat separation step. After iteration *i* in the Gauss-Newton solver, the noise weighting, *w*, in fat voxels for the next iteration *i* + 1 was recalculated as $$ {w}_{i+1}^{\ast }=\left\{\begin{array}{cc}{w}_i& \frac{\rho_i}{2{\sigma}_i}\le 1\\ {}{w}_i/\left({\rho}_i/2{\sigma}_i\right)& \frac{\rho_i}{2{\sigma}_i}>1\end{array}\right. $$, where *ρ*_*i*_ = *w*_*i*_|*f* − *d*  *Py_*i*_| is the voxel-by-voxel data term residual for the *i* th iteration, and *σ*_*i*_ is the standard deviation of *ρ*_*i*_ over all voxels.

The differential susceptibility between RV and LV blood pools (*∆χ*) was converted to blood oxygenation difference (ΔSaO_2_) using an established formula [30]:

2 $$ \Delta  {\mathrm{SaO}}_2=\frac{-\Delta  \chi }{4H{\chi}_{deoxyheme}} $$

Where *χ*_*deoxyheme*_ is the molar susceptibility of deoxyheme such that $4{\chi}_{deoxyheme}=151.054\  ppb\frac{ml}{\mu mol}$ is the molar susceptibility of a fully deoxygenated deoxyhemoglobin [39]. $H=4 Hct\frac{\uprho_{\mathrm{RBC},\mathrm{Hb}}}{M_{Hb}}$ is the heme concentration in blood (in *μmol*/*ml*), where *Hct* is the hematocrit, ${\uprho}_{\mathrm{RBC},\mathrm{Hb}}=0.34\frac{g}{ml}$ is the mass concentration of hemoglobin in a red blood cell, and ${M}_{Hb}=64450\times {10}^{-6}\frac{g}{\mu mol}$ is the molar mass of deoxyhemoglobin. For controls, *Hct* was assumed to be 47% in men and 42% in women. These values were obtained by taking the average of the range in men and in women observed in a prior study [40]. For patients, *Hct* data was obtained from peripheral blood samples. Note that the current approach only measures the susceptibility difference between RV and LV blood pools (therefore only measures the oxygen saturation difference between the RV and LV blood pools), bypassing the need to reference the blood susceptibility to a susceptibility reference (typically chosen to be water), which may or may not replicate in-vivo conditions. Post-processing was performed using MATLAB (MathWorks, Natick, Massachusetts, USA).

### QSM performance scores

Image quality (of GRE images) was assessed using two different approaches. Image quality for individual short axis slices (in-plane data) was scored semi-quantitatively based on visually assessed motion artifact/endocardial blurring (0 = severe, 1 = moderate, 2 = negligible). Scoring was performed by consensus of three experienced physicians (JWW, JK, JK). Through plane image quality was measured quantitatively based on the magnitude of slice misregistration (from both in-plane and through-plane motion), which was measured as the standard deviation of the second derivative along heart surface curves that were obtained from two perpendicular reformatted long-axis images that depict the heart-lung interface [30]: a higher standard deviation corresponded to lower through plane image quality (less smooth heart-lung interface).

### Statistical methods

Continuous variables were compared between groups using Student’s t-tests (expressed as mean ± standard deviation). 2D and 3D image quality scores were compared using a two-tailed Wilcoxon paired-sample signed rank test. Pearson correlation coefficients, Deming linear regression [41], and the Bland Altman plots were used to test the reproducibility in the two scans from the second group of healthy subjects, to test the associations between the QSM based ΔSaO_2_ and the invasively quantified ΔSaO_2_, and to test the reproducibility in scans with and without contrast. Two-sided *p* < 0.05 was deemed indicative of statistical significance. Statistical analyses were performed using MATLAB and Prism 7 (GraphPad Software, La Jolla, California, USA).

## Results

### Normative controls

3D_NAV_QSM and 2D_BH_QSM were acquired in a cohort of 10 healthy subjects (31 ± 4 y, 60% male). Whereas in-plane image quality (as visually assessed by three experienced physicians using a semi-quantitative score) was higher for 2D_BH_QSM compared to 3D_NAV_QSM (2.0 ± 0.0 vs. 1.1 ± 0.4, *p* < 0.001), through plane image quality tended to be higher (lower slice misregistration) for 3D_NAV_QSM compared to 2D_BH_QSM (1.0 ± .2 vs. 1.5 ± .8, *p* = 0.14).

**Fig. 1:**
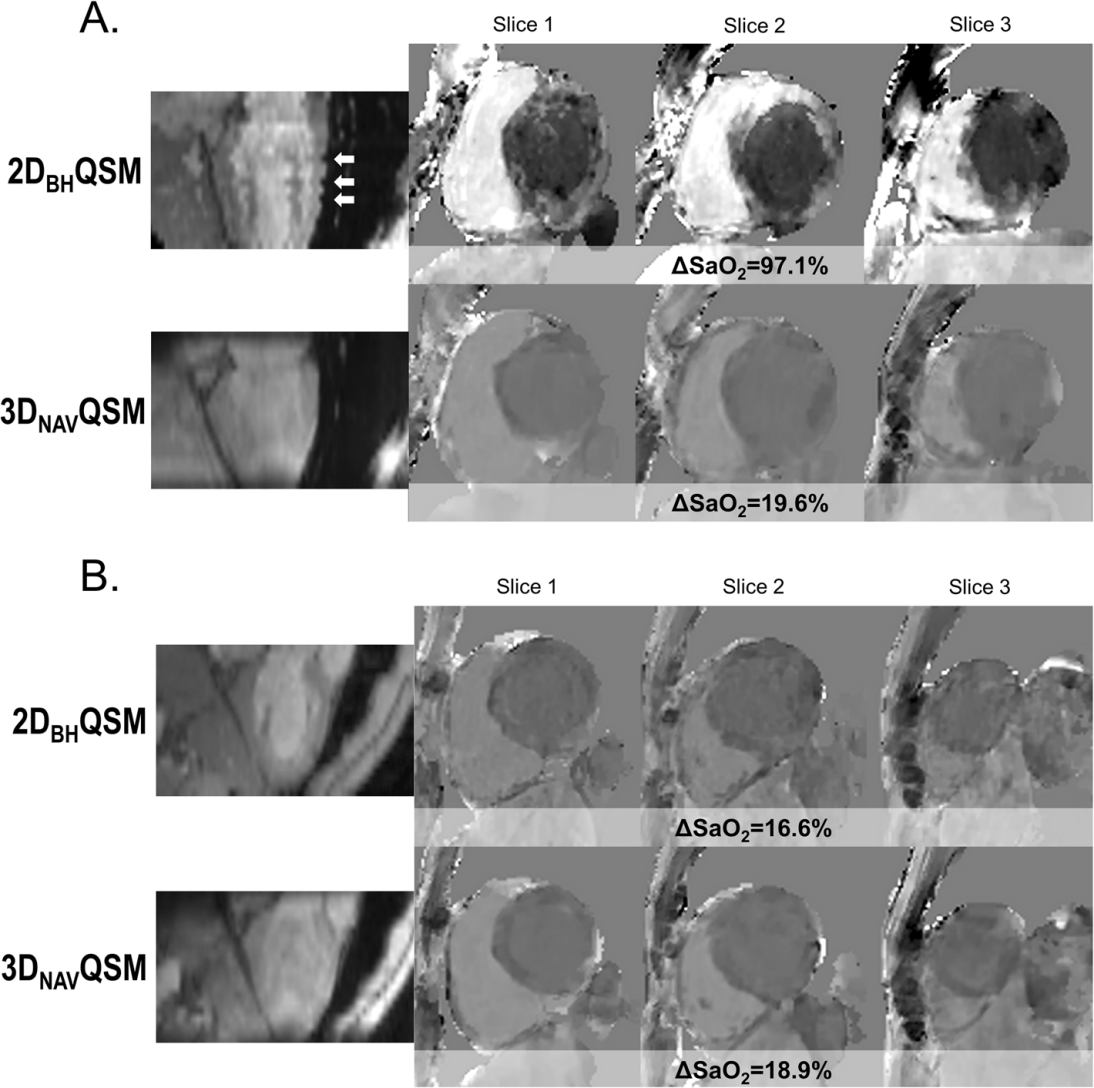
Representative Examples of Cardiac quantitative susceptibility mapping (QSM) in Healthy Subjects. **a** Unsuccessful 2D_BH_QSM due to slice mis-registration (white arrows) attributable to inconsistent breath-hold positions, resulting in non-diagnostic QSM map. Corresponding 3D_NAV_QSM was successful, yielding physiologic differential oxygen saturation between the left ventricle (LV) and right ventricle (RV). **b** Successful 2D_BH_QSM and 3D_NAV_QSM, resulting in equivalent QSM maps

3D_NAV_QSM successfully generated interpretable QSM in all 10 healthy subjects, whereas 2D_BH_QSM was successful in only 6/10 (60%) of cases. Figure 1 provides a representative example of as an interpretable QSM dataset acquired by 3D_NAV_QSM despite non-interpretable 2D_BH_QSM (**1A**), as well an interpretable QSM dataset concordantly acquired by 2D_BH_QSM and 3D_NAV_QSM (**1B)**.

In all cases for which 2D_BH_QSM was uninterpretable, failure was due to slice misregistration between sequential LV short axis datasets. Consistent with this, quantitative slice misregistration was over 2-fold higher in cases for which 2D_BH_QSM failed (*n* = 4) compared to cases (*n* = 6) in which 2D_BH_QSM yielded diagnostic results (2.3 ± 0.4 vs. 1.0 ± 0.2, *p* < 0.001). There was no significant difference in slice misregistration between 3D_NAV_QSM and 2D_BH_QSM in exams for which both sequences were successful (1.0 ± 0.1 vs 1.0 ± 0.2, *p* = 1.0).

Regarding data acquisition time, results demonstrated 3D_NAV_QSM to yield a 30% reduction compared to 2D_BH_QSM (4.7 ± 0.9 vs. 6.7 ± 0.5 min, *p* = 0.002) attributable to the interval time between each breath-hold. Navigator efficiency for 3D_NAV_QSM was 54 ± 12%. Reduced scan time yielded by 3D_NAV_QSM remained significant even among controls in whom both pulse sequences produced diagnostic results and acquisitions were reduced by an average of 37% (4.3 ± 1.4 vs. 6.8 ± 0.4 min, p = 0.002), with navigator efficiency for 3D_NAV_QSM 56 ± 16%.

Regarding QSM results, mean RV/LV susceptibility difference was not significantly different between 3D_NAV_QSM and 2D_BH_QSM acquired in controls in whom the latter pulse sequence was successful (252 ± 39 ppb vs. 211 ± 29 ppb, *p* = 0.39), corresponding to ΔSaO_2_ of 17.5 ± 3.1% and 14.7 ± 2.0%, respectively. Of note, the RV/LV susceptibility difference (and ΔSaO_2_) calculated using 3D_NAV_QSM was not significantly different when compared between controls with and without diagnostic results yielded by 2D_BH_QSM (252 ± 39 ppb vs. 250 ± 33 ppb, *p* = 0.87 [17.5 ± 3.1% vs. 17.3 ± 2.4%]).

**Fig. 2:**
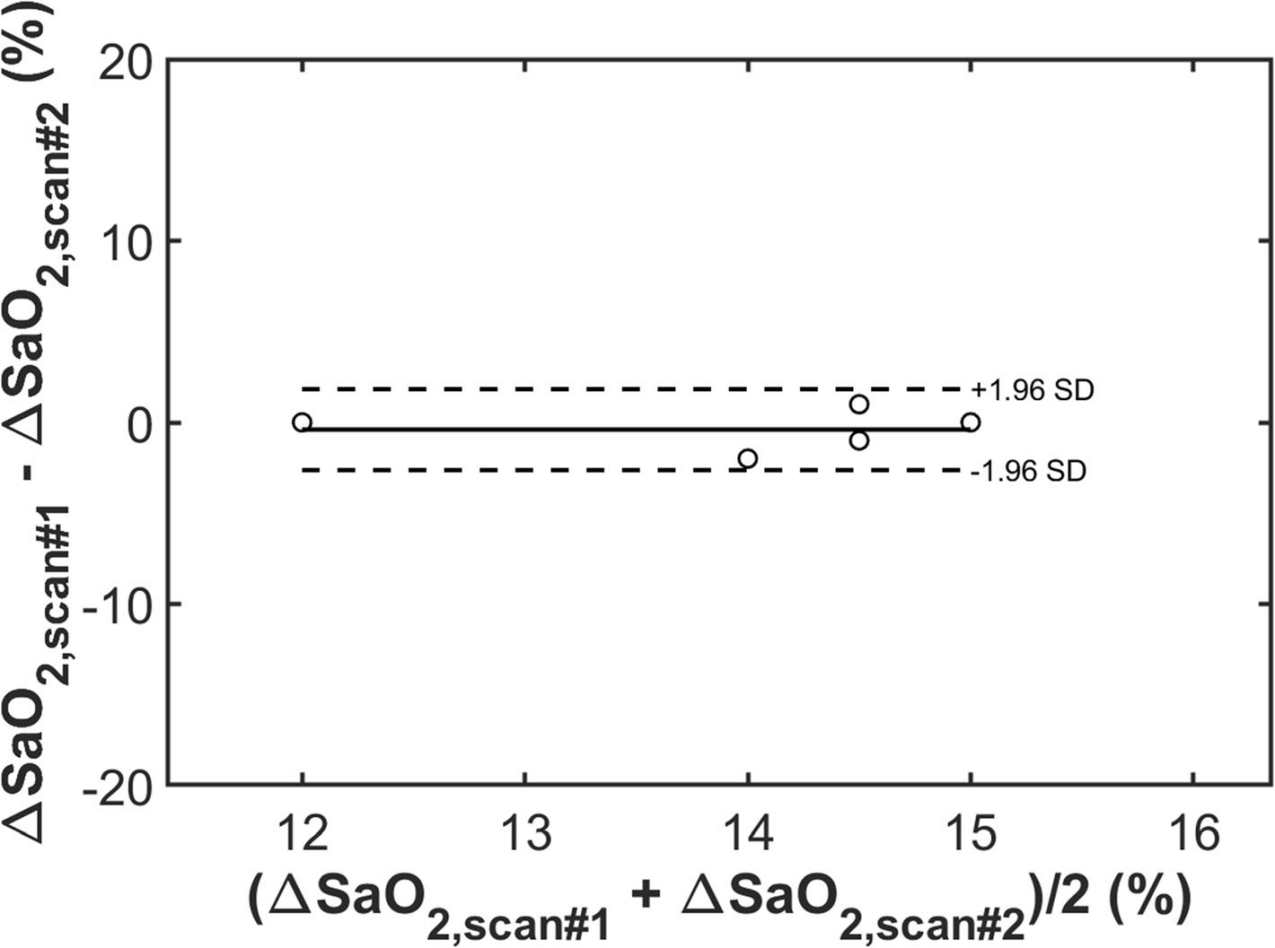
ΔSaO_2, *QSM*_ reproducibility experiment in normative controls *N* = 5 healthy controlls were scanned and rescanned to test the reproducibility of QSM based ΔSaO_2_ measurement. The ΔSaO_2_ measured by QSM between the two scans were very similar: small bias (− 0.4%) and reasonable limits of agreement (±2.2%)

Reproducibility of 3D_NAV_QSM (as tested in 5 healthy subjects) was high, as evidenced by small mean differences (− 0.4%) and reasonable limits of agreement (±2.2%) between data acquired during two separate scans (Fig. 2**)**.

### Clinical patients

3D_NAV_QSM was acquired in 39 patients whose population characteristics are shown in Table 1**.** In this group, QSM data was successfully obtained in 87% (34/39) of cases. In 5 cases, 3D_NAV_QSM yielded non-diagnostic results (non-physiological equivalence between LV and RV blood oxygenation) – all of which had substantial motion artifact. In the remainder of patients (*n* = 34), RV/LV susceptibility difference calculated using 3D_NAV_QSM was substantial (298 ± 72 ppb), corresponding to a ΔSaO_2_ of 24.9 ± 6.1% (*p* < 0.001 vs. controls). Image acquisition time in the overall clinical cohort was 6.9 ± 2.8 min; increased acquisition time tended to be longer among patients compared to controls (4.7 ± 0.9 min; *p* = 0.04) due to greater respiratory variability and lower navigator efficiency in patients (36 ± 12% vs. 54 ± 12%).

**Table 1:** Population characteristics

Age	63 ± 10yo
Gender (% male)	31% (12)
Known CAD	56% (22)
Pulmonary Hypertension	51% (20)
Atherosclerosis Risk Factors	
Tobacco Use (prior or current)	46% (18)
Hypertension	67% (26)
Hyperlipidemia	54% (21)
Diabetes mellitus	18% (7)
Medication Regimen	
ACE Inhibitors or ARB	51% (20)
Beta-Blockers	72% (28)
Aspirin	74% (29)
Statin	69% (27)
Diuretic	46% (18)
Cardiac Structure/Function	
LVEF (%)	49 ± 14%
LV Dysfunction (EF < 50%)	49% (19)
LV End-Diastolic Volume	186 ± 57 ml
LV End-Systolic Volume	106 ± 56 ml
RV EF (%)	51 ± 11%
RV Dysfunction (EF < 50%)	31% (12)
RV End-Diastolic Volume	169 ± 62 ml
RV End-Systolic Volume	96 ± 52 ml

Data reported as % (n) for categorical variables, mean standard deviation for continuous variables

*ACE* angiotensin converting enzyme, *ARB* angiotensin receptor blocker, *CAD* coronary artery disease, *EF* ejection fraction, *LV* left ventricle, *RV* right ventricular

**Fig. 3:**
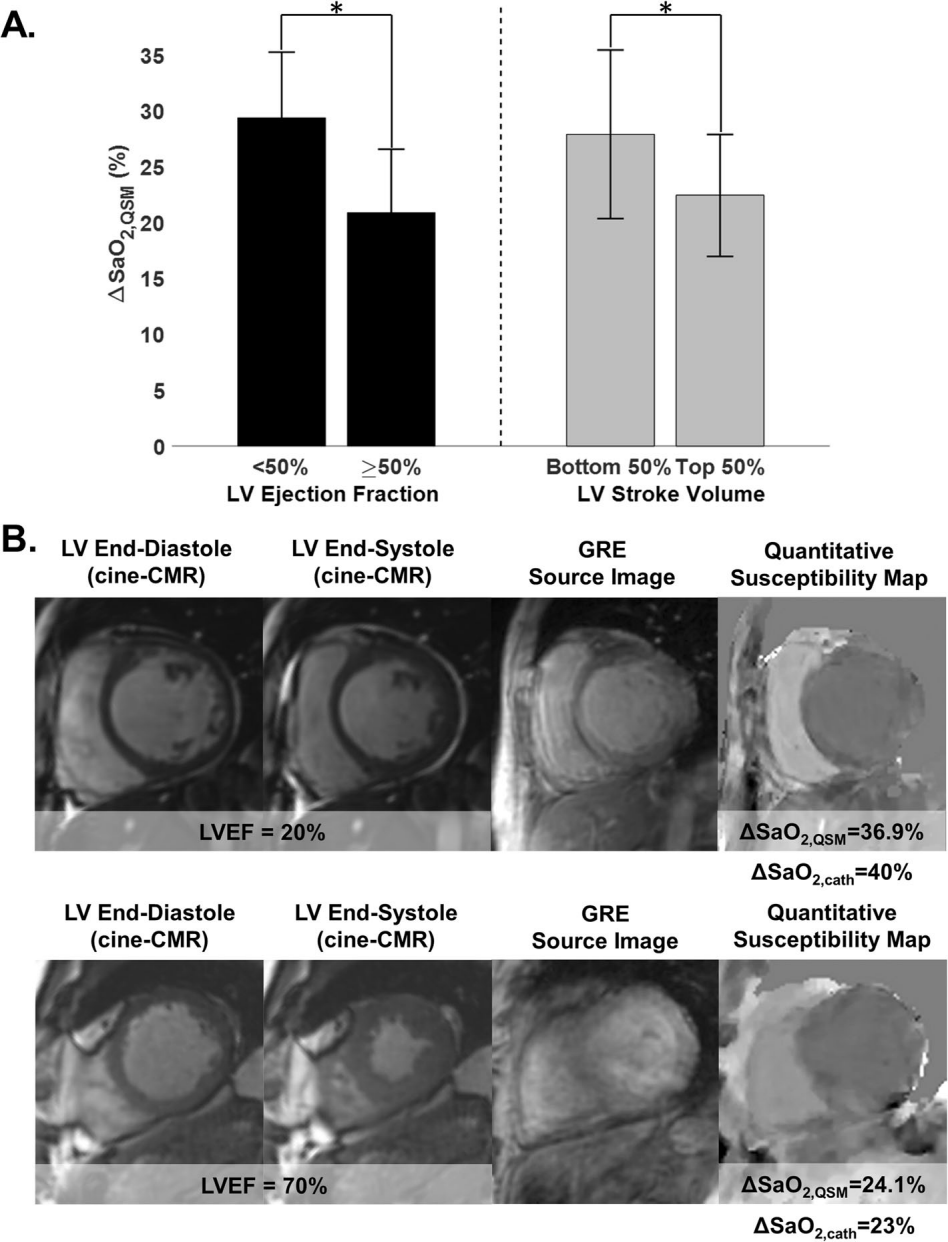
Cardiac QSM in Cardiac Patients. **a** QSM ΔSaO_2_ among patients grouped based on presence or absence of LV systolic dysfunction based on cine-CMR quantified ejection fraction (left) and stroke volume (right) (data shown as mean ± standard deviation). Note greater ΔSaO_2_ among patients with LV systolic dysfunction. **b** Two representative examples of QSM maps in cardiac patients. In the top patient, who had severely reduced LV function (EF = 20%), QSM measured a marked increase in ΔSaO_2_ (36.9%), which agreed well with invasive catheterization (40%). In the bottom patient, who had normal LV function (EF = 70%), QSM measured ΔSaO_2_ (24.1%) was within normal limits and was similar to invasive data (23%)

Among the overall clinical cohort in whom QSM was successful (n = 34), results varied in relation to LV systolic dysfunction as quantified using cine-CMR. As shown in Fig. 3a, a greater ΔSaO_2_ on QSM was observed among patients with LV dysfunction (EF < 50%) as quantified by cine-CMR (29.4 ± 5.9% vs. 20.9 ± 5.7%, *p* < 0.001). Similarly, patients in the bottom median of cine-CMR quantified LV stroke volume had greater ΔSaO_2_ on QSM (27.9 ± 7.5% vs. 22.4 ± 5.5%, *p* = 0.013).

**Fig. 4:**
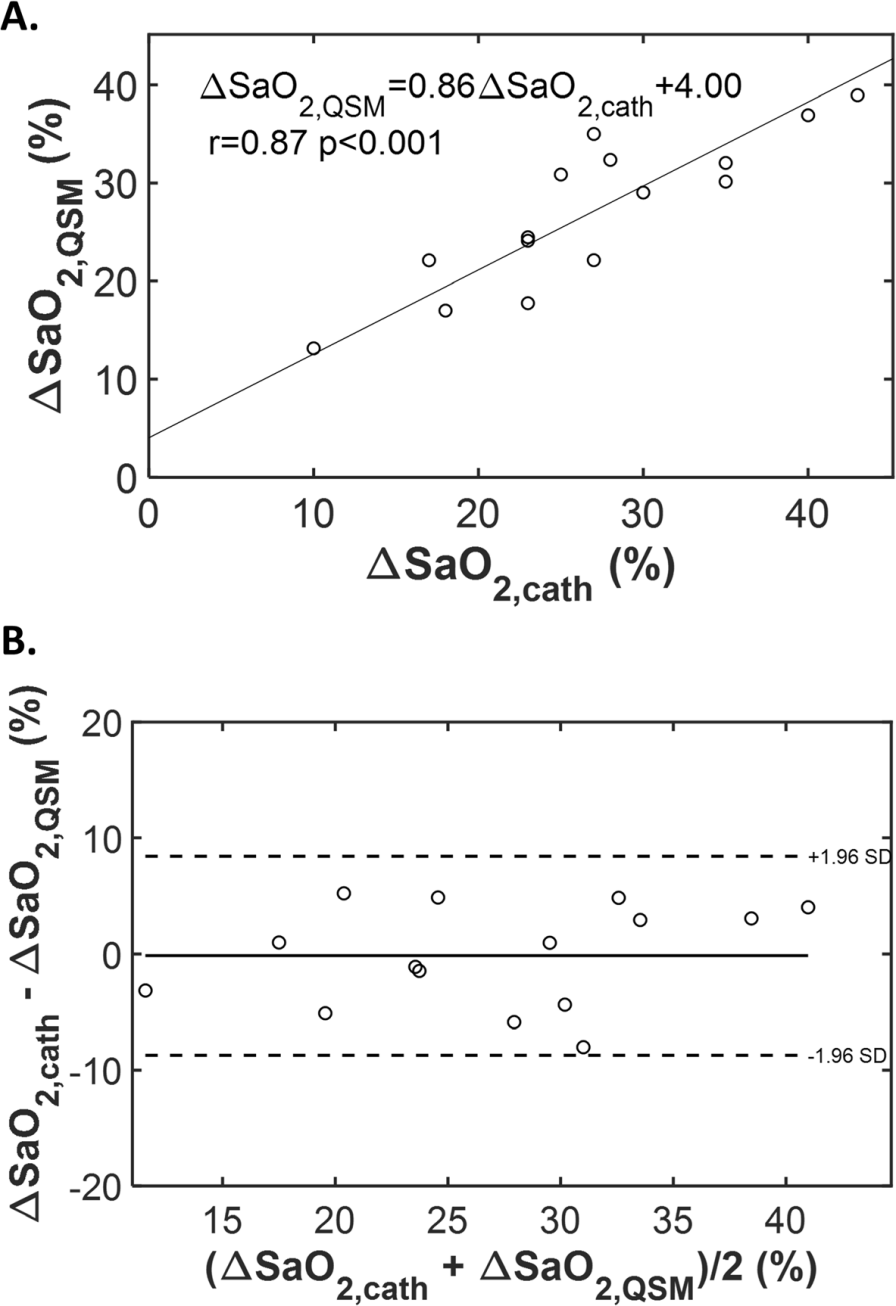
Cardiac QSM in Relation to Invasive Catheterization. **a** Scatter plot examining QSM derived ΔSaO_2_ in relation to invasive catheterization derived ΔSaO_2_. A good correlation (r = 0.87, *p* < 0.001) and linear relationship between the two approaches is observed. **b** Bland Altman plot. Note small bias between the two tests (−.1%) and moderate limits of agreement (±8.6%)

In a subgroup of 15 patients who had successful QSM, invasive cardiac catheterization (cath) was available as a reference standard for heart chamber oxygenation: all patients underwent catheterization for evaluation of known/suspected heart failure – 47% had LV systolic dysfunction and 53% had primary pulmonary hypertension. Mean interval between tests (cath, CMR) was 4 ± 3 days (range 0–12 days). Figure 3b provides representative patient examples, including close agreement with invasively quantified ΔSaO_2_ and increased magnitude of difference in context of LV dysfunction. As shown in Fig. 4a, QSM yielded good correlation with invasively quantified ΔSaO_2_ (r = 0.87, *p* < 0.001); corresponding to small bias (− 0.1%) and reasonable limits of agreement (±8.6%) between the two tests (Fig. 4b). Table 2 provides a breakdown of QSM results on a per-patient basis, together with invasive cath data and corresponding indices of LV function. Consistent with results in the overall clinical cohort, patients who underwent cath demonstrated LV systolic dysfunction (EF < 50%) to be associated with greater ΔSaO_2_ on both invasive testing (32.9 ± 3.7% vs. 21.2 ± 6.9%, *p* = 0.002) and non-invasive QSM (33.9 ± 5.6% vs. 21.2 ± 5.8%, p < 0.001).

**Table 2:** QSM in relation to Invasive Catheterization ΔSaO_2_ and Cine-CMR Cardiac Function

Patient	cath Δ SaO_2_ (%)	QSM Δ SaO_2_ (%)	LV Ejection Fraction (%)	LV Stroke Volume (ml)	LV Dysfunction (1 = EF < 50%)	Pulmonary Hypertension (1 = present)
1	25	31	66	96	0	1
2	17	22	70	60	0	0
3	23	24	71	140	0	0
4	10	13	70	90	0	0
5	35	32	47	71	1	1
6	30	29	28	77	1	0
7	40	37	20	48	1	0
8	43	39	20	60	1	1
9	27	35	16	66	1	1
10	28	32	40	48	1	1
11	27	22	66	28	0	1
12	35	30	33	47	1	1
13	23	18	65	94	0	0
14	23	24	54	126	0	0
15	18	17	69	93	0	1

### Cardiac QSM before and after contrast administration

**Fig. 5:**
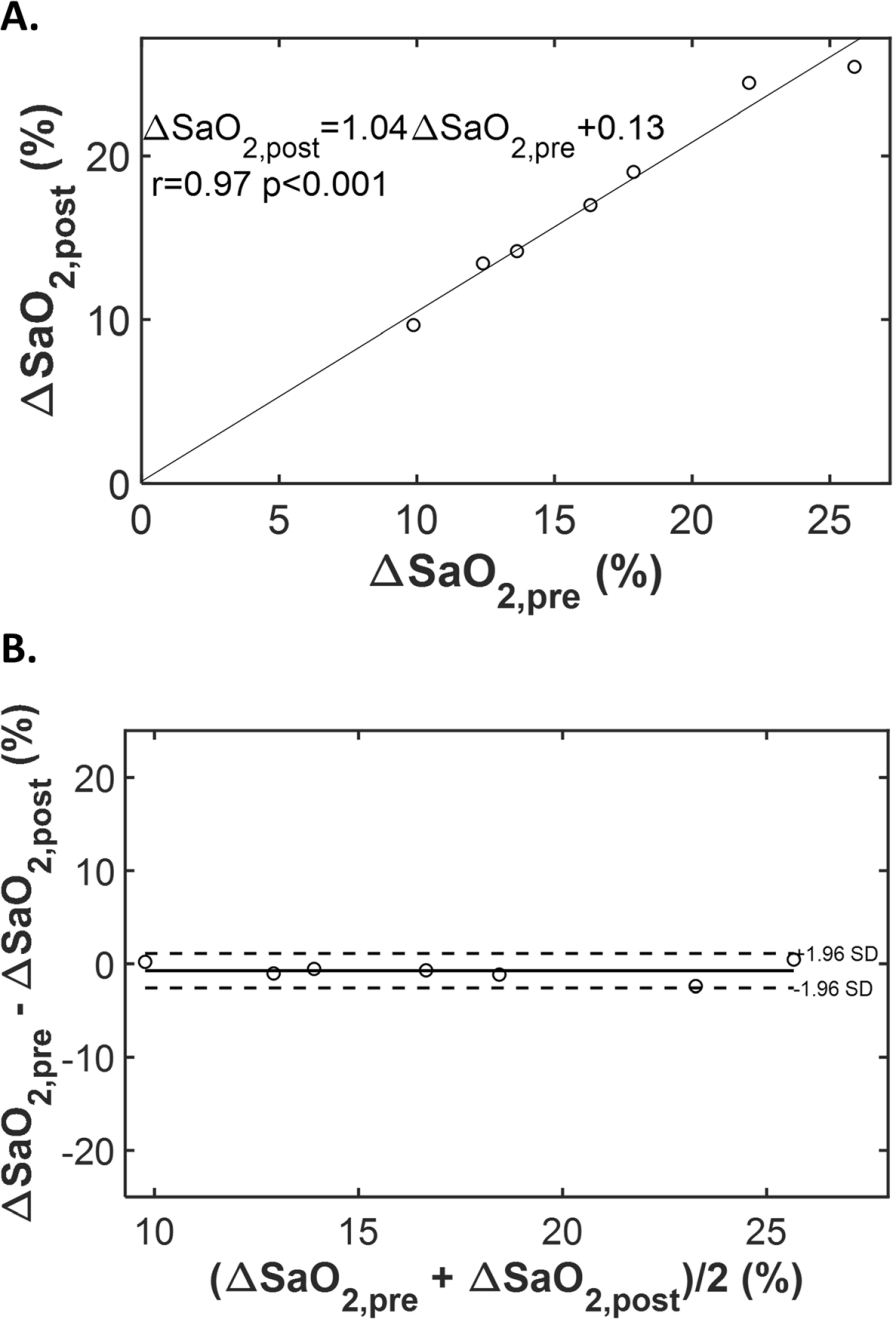
QSM based ΔSaO_2_ measured pre- and post-contrast administration. **a** Scatter plot examining QSM derived ΔSaO_2_ pre- and post-contrast administration. A good correlation and linear relationship between the two measurements is observed. **b** Bland Altman plot showing small bias (− 0.7%) and small limits of agreement (±1.9%)

3D_NAV_QSM was obtained successfully in all 7 subjects who were scanned both before and after contrast administration. As shown in Fig. 5, the ΔSaO_2_ measured from pre- and post-contrast QSM matched very well (slope = 1.04, r = 0.97, p < 0.001), with a small bias (− 0.7%), small limits of agreement (±1.9%), and a small mean absolute difference (0.9%).

## Discussion

This is the first study to test the free breathing 3D cardiac QSM for non-invasive measurement of blood oxygenation, inclusive of validation data provided by cine-CMR quantified cardiac remodeling and invasively quantified oxygen saturation in cardiac patients. Key findings are as follows: first, among a normative test cohort, 3D_NAV_QSM had a greater success for interpretable results than did 2D_BH_QSM (100% vs. 60%) and did so within shorter scan time. Second, 3D_NAV_QSM performed robustly in a subsequent cohort of 39 patients with established cardiovascular disease, among whom results demonstrated differential LV/RV susceptibility in 87% (34/39) of cases: magnitude of ΔSaO_2_ differed in relation to LV systolic dysfunction as quantified on cine-CMR, as evidenced by greater ΔSaO_2_ on QSM among patients with impaired LVEF (< 50%) compared to those with preserved LVEF, and similar results when QSM results were compared in relation to decreased LV function as stratified based on stroke volume. Third, among a subgroup of patients undergoing invasive catheterization, 3D_NAV_QSM yielded good correlation with invasively quantified ΔSaO_2_, corresponding to reasonably small bias and limits of agreement (− 0.1 and ± 8.6%, respectively) between approaches.

While our data validate cardiac QSM for differential chamber oxygenation, it is important to recognize that prior research has applied different CMR approaches for this purpose. Most previous approaches are based on measurement of blood CMR relaxation times (T2, T2*, and T1) [6–10, 12, 13, 42]. However, conventional methods based on longitudinal (T1) or transverse (T2) relaxation properties can be challenging to apply clinically. For example, the dependence of spin echo T2 on oxygenation is well understood, but in practice requires measuring several model parameters in addition to oxygenation, thereby potentially limiting accuracy or complicating clinical implementation. Recently, an oxygen saturation measurement based on acquiring multiple T2 maps using a 2D T2 prepared bSSFP sequence designed to overcome these limitations was shown to provide good agreement with invasive catheterization based measurement in an animal study. A comparison between this promising approach and our proposed QSM approach is warranted in a future study [11]. An alternative approach consists of quantifying the magnetic susceptibility of blood. The physical model relating blood susceptibility to oxygen saturation is simpler than that for T2 as it is linear with the slope a known physical constant. The magnetic susceptibility of venous blood can be computed from the CMR image phase by geometric modeling [43–47]. Mapping of magnetic susceptibility throughout the 3D field of view, as is done in QSM, enables measurement of oxygen saturation of any vascular structure (including the heart) by simple ROI analysis [25, 26, 48]. Our current data extends on prior work by our group that has shown QSM to provide an index of hemorrhage [26, 28], as well as an index of metabolism and oxygen utilization in the brain [49, 50].

Our QSM results regarding differential LV and RV blood oxygen saturation are consistent with values reported in prior literature as well as expected differences between subjects with and without cardiovascular disease. Regarding control data, ΔSaO_2_ measured from 3D_NAV_QSM (17.5 ± 3.1%) was in agreement with a prior study that reported ΔSaO_2_ in healthy subjects undergoing invasive cardiac catheterization [51], in which a mean difference of 18.8% was reported (arterial: 97.3%, venous: 78.5%). ΔSaO_2_ as measured in controls were also lower than that in patients with cardiovascular disease (17.5 ± 3.1% vs. 24.9 ± 6.1%, *p* < 0.001), consistent with expected physiological differences between the two groups. Among our subgroup of patients with cath validation (*n* = 15), QSM derived ΔSaO_2_ demonstrated a linear relationship with invasive measurements, and bias between the two oxygenation measurement approaches was small. While our data cannot be interpreted in context of an exact partition value with which to guide clinical decision making, it should be noted that an array of studies have demonstrated prognosis to vary in relation to magnitude of differential oxygen saturations between the left and right heart [1–5]. In this context, our findings suggest promise for development of QSM derived ΔSaO_2_ as a non-invasive risk stratification tool, such that clinically stable patients with normal values would be screened out as low risk, whereas those with elevated QSM derived ΔSaO_2_ are referred for confirmatory invasive testing.

Note that, in this work, QSM was performed during non-contrast imaging in healthy subjects, and at the end of contrast-enhanced exams (~ 30 min post gadolinium infusion) in patients. Whereas gadolinium changes blood susceptibility, prior data has shown gadolinium to be near completely cleared from myocardium, and to be well mixed in the intravascular space at our imaging time point [52, 53]. Accordingly, QSM calculations were based on the premise that contrast blood pool concentration was constant across cardiac chambers, such that gadolinium contributions to susceptibility in the LV and RV cancel out when computing differential oxygenation. Indeed, our data demonstrate that cardiac QSM derived ΔSaO_2_ measurements agree well between pre- and post-contrast.

One key technical innovation in our current study concerns use of navigator technology for free breathing 3D QSM. The conventional cardiac 2D_BH_QSM approach generates data by imaging LV (short axis) slices individually via sequential breath-holds in order to reconstruct a 3D field map. When one or more of these breath-holds is acquired at a different respiratory position than the others, the resulting field map will not be a true 3D volume, and the QSM map will contain artifacts as the reconstruction model assumes a continuous 3D dataset as input. The susceptibility inversion problem is inherently 3D, given that susceptibility changes within one region of a given structure (e.g. RV blood pool) affects the field in the surrounding areas in all three (x, y, z) spatial directions: this is because the field is a 3D convolution of the underlying 3D susceptibility distribution with the dipole kernel. The 3D_NAV_QSM approach has no inherent mis-registration limitation, and the success of 3D_NAV_QSM acquisition depends on navigator accuracy in motion tracking. These aspects of QSM reconstruction may explain our seemingly discordant finding regarding GRE image quality and diagnostic performance of the two QSM pulse sequences tested in our study: even though GRE images from the 2D_BH_QSM sequence were assigned higher image scores than images from the 3D_NAV_QSM sequence, 2D_BH_QSM failed to generate interpretable QSM more often than did 3D_NAV_QSM and this failure was primarily attributable to slice mis-registration.

There are several limitations of our study. First, scan time of 3D_NAV_QSM sequence is long (~ 4–7 min) compared to other routine clinical sequences. One potential approach to shorten scan time is to increase parallel acceleration factor, use compressed sensing [54, 55], and/or apply data acquisition strategies such as echo planar readout [56] or echo sharing methods [57]. Non-Cartesian acquisitions that allow for self-gating [58] and multi-phase reconstruction [59] are also alternatives to shorten cardiac QSM. A second limitation to the current 3D_NAV_QSM approach is that the respiratory motion is tracked by a diaphragmatic navigator, which is known to occasionally fail [60]. More advanced navigator techniques such as a fat navigator can be used to improve success rates [31, 32, 61–63]. A third issue to consider is that the QSM validation in this study was derived from cardiac remodeling indices in 39 cardiac patients of whom 15 had invasive catheterization for evaluation of known or suspected heart failure; further validation in a larger clinical cohort including patients undergoing catheterization for different indications is warranted. Finally, whereas the interval between QSM and invasive catheterization was relatively short (mean 4 ± 3 days, median 2 days [IQR 1–5 days]), cardiac chamber oxygenation could have varied during this time, thus resulting in discordance between tests. Future research with simultaneous invasive and CMR oxygenation measurements in animal models could be of utility for further validation of QSM, as well as comparisons with alternative pulse sequences for oxygenation quantification (i.e. T2). Nevertheless, our current findings that 3D navigator QSM was feasible among a clinical cohort in whom it generally agreed with differential oxygenation saturation as measured invasively is of substantial importance with respect to translational application of this technique, and provides initial proof of concept with respect to clinical implementation.

Heart rate could have impacted our QSM results. This has been shown to be the case for oxygenation methods such as BOLD, which relies on the magnitude of the CMR signal. On the other hand, QSM, which relies on the phase of the CMR signal, is expected to be relatively insensitive to heart rate because the contributions to the phase (field) induced by the difference in LV and RV magnetic susceptibility (oxygenation) are not affected by heart rate. Further research is warranted to specifically assess physiologic factors impacting QSM, as well as to validate this pulse sequence in a larger clinical cohort including patients undergoing catheterization for different indications.

In conclusion, we provide validation of free breathing 3D cardiac QSM as an index of cine-CMR evidenced LV dysfunction and differential LV/RV oxygen saturation as measured by invasive catheterization. Future research is necessary to test accelerated free-breathing QSM strategies, refine cardiac QSM for myocardial tissue characterization, and validate QSM-derived blood oxygenation for non-invasive stratification of heart failure symptoms and prognostic outcomes.

## Data Availability

The datasets generated and/or analyzed during the current study are available from the corresponding author on reasonable request.

## Abbreviations

2D_BH_QSM: 2D Breath-hold QSM approach
3D_NAV_QSM: 3D free-breathing QSM approach
bSSFP: balanced steady state free precession
cath: Catheterization
CMR: Cardiovascular magnetic resonance
ECG: Electrocardiogram
EF: Ejection fraction
GRE: Gradient recalled echo
Hct: Hematocrit
IDEAL: Iterative decomposition of water and fat with echo asymmetry and least squares estimation
LV: Left ventricle/left ventricular
MEDI: Morphology enabled dipole inversion
MERIT: An iterative reweighted least squares fitting method
O_2_: Oxygen
PAWS: Phase-ordered automatic window selection
QSM: Quantitative susceptibility mapping
RHC: Right heart catheterization
ROI: Region of interest
RV: Right ventricle/right ventricular
SNR: Signal-to-noise ratio
TE: Echo time
TR: Repetition time
ΔSaO_2_: Differential blood oxygenation between the left and right heart
